# The 3′ Splice Site of Influenza A Segment 7 mRNA Can Exist in Two Conformations: A Pseudoknot and a Hairpin

**DOI:** 10.1371/journal.pone.0038323

**Published:** 2012-06-07

**Authors:** Walter N. Moss, Lumbini I. Dela-Moss, Elzbieta Kierzek, Ryszard Kierzek, Salvatore F. Priore, Douglas H. Turner

**Affiliations:** 1 Department of Chemistry, Center for RNA Biology, University of Rochester, Rochester, New York, United States of America; 2 Institute of Bioorganic Chemistry, Polish Academy of Sciences, Poznan, Noskowskiego, Poland; University of Florida, United States of America

## Abstract

The 3′ splice site of influenza A segment 7 is used to produce mRNA for the M2 ion-channel protein, which is critical to the formation of viable influenza virions. Native gel analysis, enzymatic/chemical structure probing, and oligonucleotide binding studies of a 63 nt fragment, containing the 3′ splice site, key residues of an SF2/ASF splicing factor binding site, and a polypyrimidine tract, provide evidence for an equilibrium between pseudoknot and hairpin structures. This equilibrium is sensitive to multivalent cations, and can be forced towards the pseudoknot by addition of 5 mM cobalt hexammine. In the two conformations, the splice site and other functional elements exist in very different structural environments. In particular, the splice site is sequestered in the middle of a double helix in the pseudoknot conformation, while in the hairpin it resides in a two-by-two nucleotide internal loop. The results suggest that segment 7 mRNA splicing can be controlled by a conformational switch that exposes or hides the splice site.

## Introduction

Pandemic outbreaks of influenza A were responsible for millions of deaths in the 20^th^ century. Notably, the Spanish Flu of 1918 killed between 20 [1] and 100 million people [2]. Influenza is still of grave concern to public health. Each year globally there are an estimated three to five million severe infections with up to 500,000 deaths [3]; in the U.S. alone there are approximately 200,000 hospitalizations and 36,000 deaths yearly [4], [5], [6]. Most therapeutics target influenza proteins: e.g. blocking the M2 ion channel with amantadine and rimantadine [7]). The virus, however, utilizes RNA at every step in its propagation, making viral RNA an attractive target for therapeutic treatment [8], [9]. A better understanding of the structure and function of RNA in influenza A would open new avenues for treatment of this deadly disease, and provide a valuable complement to current therapeutics.

**Figure 1:**
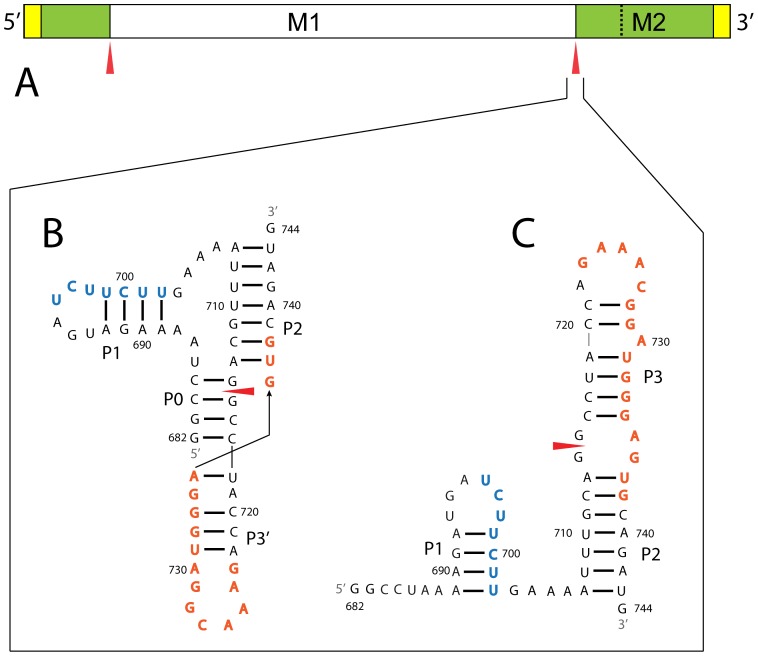
Location and structure of the 3′ splice site. (**A**) Segment 7 mRNA annotated with the splice sites (red arrow), UTRs (yellow box), and M1 and M2 open reading frames. M1 and M2 share the same start site, but M1 stops within the M2 ORF (at the black dotted line). Splicing combines the two green boxes to form the M2 open reading frame. (**B**) Predicted pseudoknot (PK) model with 3′ splice site annotation (red arrow), an SF2/ASF exonic splicing enhancer binding site in orange, and a polypyrimidine tract in blue. (**C**) Predicted hairpin (HP) model with same annotations as B. In order to isolate a single conformation, specific mutations were introduced to change nts 684 to 686 into A’s and to swap GC for CG at pairs 716–734 and 717–733 to forbid the pseudoknot and only allow the hairpin fold (HPMut).

The influenza A virus possesses an eight segment (–) sense RNA genome, which codes for at least eleven proteins. Fragments of the influenza A coding RNA are predicted to have unusual thermodynamic stability, and also have suppressed third codon position variability. In combination with conserved base pairing, these results provided predictions of fragments likely to fold into functional structures [10]. One particularly interesting fragment (Fig. 1) includes the 3′ splice site of segment 7, as well as key residues of a binding site for the human SF2/ASF splicing factor [11] and a polypyrimidine tract that may bind other splicing factors such as U2AF65 [12], [13]. Segment 7 encodes the M1 matrix protein and three alternatively spliced products that share the 3′ splice site: the M2 protein, the small M3 polypeptide, and occasionally M4 [14]. Production of M2 is critical for uncoating of the viral genome and splicing of the M2 mRNA is temporally controlled [15].

Secondary structure modeling of the 3′ splice site of segment 7 (Fig. 1) yielded the possibility of two alternative conformations: (1) a pseudoknot (Fig. 1B), where the splice site is base paired in a helix and (2) a hairpin (Fig. 1C), where the splice site occurs in a two-by-two nt internal loop [10]. Native gel analysis, enzymatic/chemical structure probing, and oligonucleotide binding studies reported here for a 63 nt fragment are consistent with these models. A similar hairpin/pseudoknot was described for the 3′ splice site of segment 8, which was proposed to influence splicing of the NS2 mRNA [16], [17]. These results suggest that splicing of segment 7 may be modulated by varying splice site accessibility [18], [19], [20], [21] or splicing factor binding [22], [23], [24], and that conformational switching may be a common mechanism to control splicing of influenza genes. Small molecules [25], [26], [27], [28], [29] or oligonucleotides [30], [31], [32] that specifically bind to these structures could be used to test their function and potentially provide leads for therapeutics.

## Results

### Native Gel Electrophoresis Reveals Two Conformations

**Figure 2:**
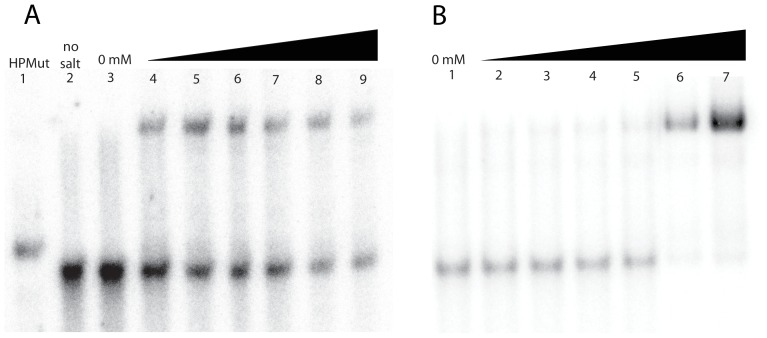
Native gels of 3PSS and HPMut under various conditions. (**A**) First lane is HPMut folded with 10 mM Tris (pH7), 100 mM KCl, and 10 mM MgCl_2_; the second lane is 3PSS with 10 mM Tris (pH7) and no monovalent or divalent ions. The remaining lanes are for 3PSS with 10 mM Tris (pH7) and 100 mM KCl with increasing Mg^2+^ concentration (0, 2.5, 5.0, 7.5, 10, 15, and 25 mM). (**B**) First lane is 3PSS with 10 mM Tris (pH7), 100 mM KCl and no multivalent ions. The remaining lanes are 3PSS with 10 mM Tris (pH7) and 100 mM KCl with increasing [Co(NH_3_)_6_]^3+^ concentration (0.002, 0.01, 0.02, 0.1, 2.5, and 5 mM).

Native gels were run with a 63 nt fragment of an avian influenza A 3′ splice site (3PSS) from segment 7, alongside an artificial mutant construct (HPMut) that can fold into a hairpin but not a pseudoknot. Specifically, in HPMut, nucleotides 684-6 are changed to adenosines (Fig. 1B) and the two hairpin CG pairs at nucleotides 716–734 and 717–733 are swapped to make GC pairs (Fig. 1C); both changes forbid formation of the pseudoknot P0 helix while maintaining the hairpin. When the wild type sequence is folded in the presence of Mg^2+^, two bands are observed (Fig. 2A). The faster running of the two major bands observed for 3PSS (lanes 4–9 of Fig. 2A) migrates similar to HPMut (lane 1 of Fig. 2A). This suggests that the faster running band is the hairpin (HP) conformation of 3PSS and, by exclusion, that the slower running band is the pseudoknot (PK) conformation.

In the absence of multivalent cations there is no observable amount of PK (Fig. 2A lane 3; 2B lane 1). In the presence of Mg^2+^, 3PSS folds into both HP and PK conformations (Fig. 2A) but even with 25 mM Mg^2+^ PK never becomes dominant (Fig. 2A lane 9). However, when 3PSS is incubated with 5 mM cobalt (III) hexammine ([Co(NH_3_)_6_]^3+^), the dominant product becomes PK (Fig. 2B, lane 7). Henceforth, 3PSS will be referred to as PK when in the presence of 5 mM [Co(NH_3_)_6_]^3+^ and as HP when in the presence of only 100 mM KCl.

### Enzymatic and Chemical Mapping of RNA Secondary Structure

**Figure 3:**
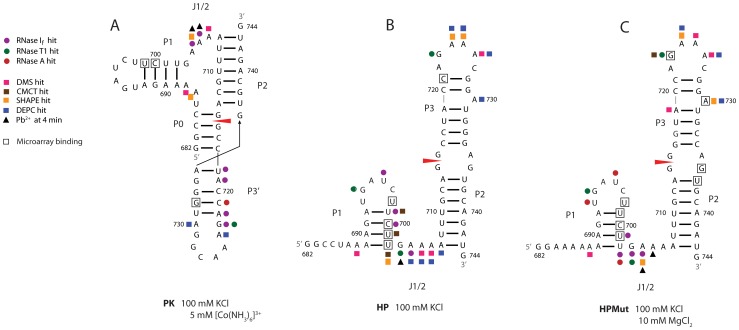
Mapping results for 3PSS and HPMut. The reagents are indicated by shape and color (see figure key). Only strong hits, with ≥2/3 the highest intensity band in a lane after subtraction of intensity from the control lane, are annotated. Pb^2+^ mapping results are taken from 4 min incubation time. For Pb^2+^, strong hits had ≥2/3 the intensity of A704 from PK, the strongest band observed under any condition at 4 min incubation time. Boxed residues are the center nucleotide of sites binding strongly (≥1/3 the highest intensity spot) and unambiguously to microarray probes (i.e. without alternative binding sites capable of forming stable duplexes within 3 kcal/mol of the optimal binding site). All folding buffers contained 10 mM Tris (pH7), 100 mM KCl. (**A**) Mapping results for 3PSS folded in 5 mM [Co(NH_3_)_6_]^3+^ (PK). (**B**) Mapping results for 3PSS folded without Mg^2+^ or [Co(NH_3_)_6_]^3+^ (HP). RNase A reactivity is not annotated because RNA is over-digested at the same enzyme concentration that yielded good results in PK and HPMut (Fig. S3). (**C**) Mapping results for HPMut folded in 10 mM Mg^2+^.

To study individual conformations, mapping was carried out on 3PSS with 100 mM KCl and no multivalent cations to favor HP, and with 100 mM KCl and 5 mM [Co(NH_3_)_6_]^3+^ to favor PK. HPMut was mapped in 100 mM KCl with 10 mM MgCl_2_. PK and HP/HPMut share structural motifs P1, P2, and junction J1/2 (Fig. 3). The differences between the two conformations are the P0 and P3′ motifs in PK and the P3 stem-loop in HP/HPMut. Enzymatic mapping used RNase T1 (cleaves after unpaired G), RNase A (cleaves after unpaired C and U), and RNase I_f_ (cleaves after any single stranded nucleotide). Chemical mapping used DMS (methylates N1 of A and N3 of C when unpaired), CMCT (modifies N3 of U and N1 of G when unpaired), and DEPC (modifies an exposed N7 of A) [33]. Pb^2+^ cleavage [34], [35] and SHAPE mapping [36] were used to identify flexible regions.

**Figure 4:**
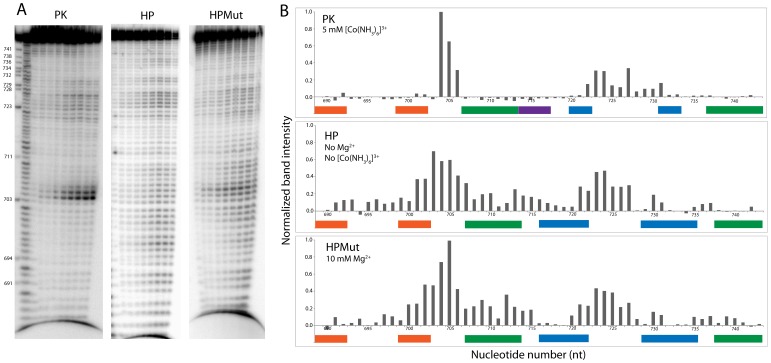
Pb^2+^ mapping results. (**A**) Gel images of PK (5 mM [Co(NH_3_)_6_]^3+^), HP (no multivalent ions), and HPMut (10 mM Mg^2+^) incubated with 1 mM Pb(OAc)_2_, 10 mM Tris (pH 7), and 100 mM KCl for 0, 0.5, 1, 2, 4, 8, 15, 30, and 60 min. (**B**) integrated band densities at each nucleotide normalized to the strongest band observed under any condition (A704 from PK) at 4 min incubation time. RNA structure is annotated below each graph where paired nucleotides that form complementary helical strands are indicated by colored boxes.

In 5 mM [Co(NH_3_)_6_]^3+^, where PK dominates, the P0 stem region, which incorporates the 3′ splice site, is not reactive (Fig. 3A). Of the three nucleotides that bridge P0 and P1, A687 is modified by SHAPE and A688 is modified by DMS. There are no strong hits on the P1 hairpin. Reactivity is clustered in the J1/2 junction and the 5′ end of the P3′ hairpin. The J1/2 junction is cleaved by RNase I_f_, Pb^2+^ and modified by SHAPE and DMS. The P3′ hairpin is quite sensitive to enzymatic cleavage, but less so towards small molecules. RNases I_f_, T1, and A cleave along the 5′ end of P3′, whereas the loop region is modified by DEPC at A724 and A730. PK is largely protected from Pb^2+^ cleavage (Fig. 4A,B). After incubation in Pb(OAc)_2_ for 4 min, strong cleavage only occurs at the J1/2 junction, particularly at A704 (Fig. 4B). Medium cleavage occurs at the loop of P3′ (Fig. 4 and Fig. S1A). Even after 60 min of incubation, PK is un-reactive outside these regions (Fig. 4A,B).

In the absence of multivalent cations, where HP dominates, P1 has many enzymatic cleavages and small molecule modification sites (Fig. 3B and S1). The J1/2 junction of HP is reactive with most reagents, particularly including DEPC. The loop of the P3 hairpin is sensitive to RNase T1, DMS, SHAPE, and DEPC. There is a strong DEPC modification at the A730 bulge. After a 4 min incubation in Pb(OAc)_2_, HP has a strong Pb^2+^ cleavage after G703 in J1/2 (Figs. 3B and 4B). After 8 min of incubation in Pb(OAc)_2_, HP is cleaved at nearly every nucleotide (Fig. 4A). Though reactivity with Pb^2+^ is widespread, the most intense cleavage (G703) has only about 67% the highest intensity observed in PK or HPMut (Fig. 4B).

3PSS folded in 10 mM Mg^2+^ yields both HP and PK in roughly equal amounts (Fig. 2A lane 7). 3PSS folded under these conditions was also probed (Fig. S1E). The reactivity observed under these conditions is consistent with both PK and HP being present. This, along with the native gel analysis, verifies the presence of both conformations when 3PSS folds in the presence of Mg^2+^.

To further test the presumed hairpin secondary structure, mapping was also carried out on HPMut in the presence of 10 mM Mg^2+^ (Fig. 3C). The P1 hairpin has strong RNases I_f_, T1, and A cleavage sites. There is also a DMS modification at A688, similar to HP. The J1/2 junction has RNases I_f_ and T1 cleavages and SHAPE and Pb^2+^ modification sites. A719 in the AU pair flanking the A730 bulge is modified by DMS. The loop of P3 is modified strongly by DMS, CMCT, SHAPE, and DEPC and also cleaved by RNase T1. SHAPE and DMS modify the A730 bulge. When normalized by the most intense cleavage in PK (A704), the Pb^2+^ cleavage pattern for HPMut is similar to that seen for HP, but the single dominant cleavage is at A705 (Figs. 3C and 4B). After 15 min of incubating in Pb(OAc)_2_, HPMut is cleaved at nearly every nucleotide (Fig. 4A). HPMut folded in 100 mM KCl and 5 mM [Co(NH_3_)_6_]^3+^was also probed with RNAse A, T1, and Pb^2+^; results were similar to those with 100 mM KCl and 10 mM Mg^2+^ (Fig. S1C).

### Accessibility to Short Oligonucleotides

PK, HP, and HPMut were hybridized to microarrays with 861 chimeric pentamer and hexamer oligonucleotides containing 2′-O-methyl RNAs with locked nucleic acid (LNA) and 2,6-diaminopurine modifications at selected positions to roughly equalize the thermodynamic stability of hybridization to unstructured RNA [37], [38], [39], [40]. Results for probes with five consecutive nucleotides Watson-Crick complementary to 3PSS are listed in Table S1. Hairpin P1 is strongly bound under all conditions tested (Fig. 3). The probes invade more of the P1 stem in HP and HPMut than in PK, however. PK also strongly binds to a probe that invades the P3′ helix (Fig. 3A). HPMut strongly binds to probes centered at the bulged A730 of P3 (Fig. 3C) and at G736 and U737 on one side of the internal loop that contains the 3′ splice site.

### Secondary Structure is Conserved

Based on the structure probing experiments and the expanded sequence alignment used in this study (Table S2), small changes are proposed for the model versus the one previously proposed [10]. The changes in the structures are shifting of nucleotides in P1 (the bioinformatics model had C700 bulged) and the revised pairing of A713 to U737 in the pseudoknot, which grew P2 at the expense of P0 (Fig. 1). A base pair is also added between U718 and A735 in P3′. The hairpin and pseudoknot were initially presented as alternative secondary structures, but both are observed in solution (Fig. 2).

**Figure 5:**
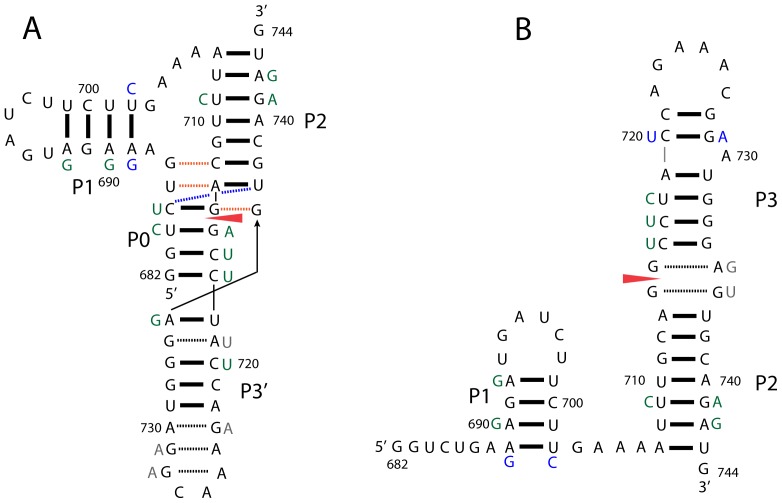
Consensus sequence and structure. (**A**) PK and (**B**) HP. Canonical pairs are indicated with solid bars and putative non-canonical pairs with dashed lines. Stem mutations that preserve base pairing are colored green for single (consistent) point mutations and blue for double (compensatory) point mutations when they occur in five or more sequences. Mutations with implications for non-canonical pairs are indicated in grey. Potential base triples are indicated with orange dashed lines. The exact interaction between the base pair and loop residue, however, cannot be inferred from available data. Putative helical stacking is indicated with a blue dashed line.

Both conformations of the 3′ splice site are well conserved throughout the alignment of all unique Influenza A sequences. All helices are greater than 92% conserved and canonical pairing, on average, is 95% conserved (Table S2). Every helix has at least one consistent or compensatory mutation (Fig. 5). When mutations led to non-canonical pairs, they were most often CA (2.9%) or GA pairs (1.3%). These CA and GA pairs occur mainly in the middle of helices: e.g. pair 691–700 in P1, 711–739 in P2, and 720–729 in P3 (Fig. 5, Table S2). Other types of non-canonical pairs were rarely observed (Table S2).

### Base Pair and GC Pair Content Correlates with Host Species

**Figure 6:**
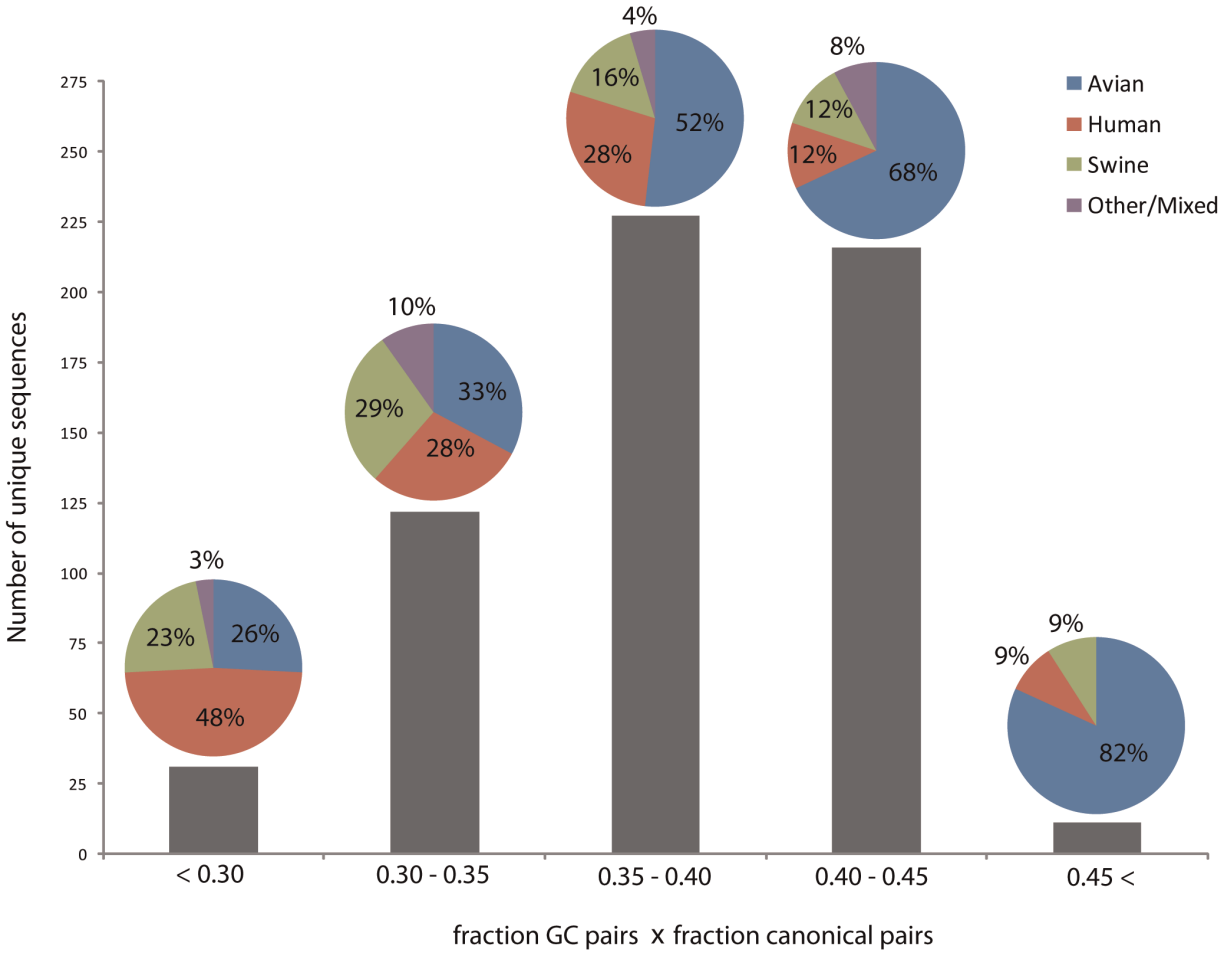
Histogram of all unique influenza A sequences grouped by expected stability of pseudoknot mutations. The stability metric is calculated as the fraction of nucleotides that are canonically paired, multiplied by the fraction of GC pairs. Above each bar is a pie chart that gives the percentage of sequences that infect a given host. Other/Mixed strains are those where the majority of sequences infected an animal from other than human, swine, or avian species; or where they could infect more than one type of host species.

On average, the pseudoknot structures expected to be most stable have 18 canonical base pairs, 50% of which are GC pairs (Table S3). The structures expected to be least stable have, on average, 16 canonical base pairs and only 31% of them are GC pairs. When clustered by the fraction of GC pairs and canonical pairs, five groups are apparent (Fig. 6). The number of unique sequences that fall within each cluster follows a bell-shaped distribution (Fig. 6). The stable clusters have sequences that allow for a greater number of canonical pairs and, in particular, GC pairs. For example, across all unique sequences, positions 684 and 715 are most often UG pairs, but in the stable clusters they are primarily CG pairs. Conversely, the less stable clusters are comprised of sequences that do not allow for as many canonical and GC pairs. Positions 711 and 739, for example, most often form a GC pair; in the less stable strains, however, these positions are mostly AC pairs (Table S2). The hairpin structure metrics globally follow that of the pseudoknot (Table S3).

The fraction of GC and canonical pairs in the pseudoknot and hairpin structures correlates with the fraction of influenza A sequences that infect a given host species (Fig. 6 and Table S3). Avian strains make up 82% of the most GC and base pair rich sequences, but only 26% of the sequences with lower content. The opposite trend holds for sequences that infect humans and swine: only 9% of the most GC and base pair rich sequences infect humans (another 9% for swine), while 48% of the sequences with lower base pair and GC content are human specific (23% are swine specific; Fig. 6).

## Discussion

Influenza remains a public health problem [41] and is also a potential agent for bioterrorism [42], [43], [44]. Current therapeutics target influenza proteins [45]. RNA, which is used throughout influenza replication, is an attractive alternative target. The results presented here show that a fragment of influenza A segment 7 can fold into two different conformations: a hairpin, HP, and a pseudoknot, PK. The predicted free energies of HP and PK are −16.9 kcal/mol and −16.3 kcal/mol, respectively. This small difference in free energy predicts that the equilibrium constant for the two conformations is close to 1. Both conformations have significant concentrations when Mg^2+^ is present (Fig. 2), consistent with the predicted free energy difference between them. Stabilizing the free energy by as little as 1.5 kcal/mol, which can be supplied by one or two hydrogen bonds, will shift the equilibrium constant by a factor of 10. Thus, small perturbations, such as protein binding, can push the equilibrium to one conformation. Switching between HP and PK may have a role in the regulation of splicing and this suggests that these RNA structures have potential as therapeutic targets.

### Conformational Switching has Implications for Function

The presence of two conformations has implications for function, as each conformation places several functional elements in different structural contexts. In 3PSS, the pseudoknot may make the sequences required for molecular recognition inaccessible to splicing elements; whereas, in the hairpin conformation, these elements are less constrained by structure and are presumably better able to interact with splicing factors. The most obvious case is sequestration of the splice site in P0 of PK versus exposing it in a two-by-two nt internal loop in HP. A similar equilibrium between hairpin and pseudoknot structures occurs within the 3′ splice site of segment 8 of influenza A and B and has been proposed to play a role in the regulation of splicing [16], [17]. The segment 8 structures are different, but also place the splice site in paired or unpaired structural contexts, potentially modulating splicing by hiding or revealing the splice site. This may be a common mechanism to control splicing: For example, sequestration of cryptic 3′ splice sites in pseudoknots of the yeast actin mRNA [46] and Sd.cob,1 group I intron [47] acts to suppress splicing at these sites.

The region surrounding the 3′ splice site of segment 7 also contains the key residues of the SF2/ASF exonic splicing enhancer binding site in the purine rich stretch from nucleotides 723 to 738 [11]. These residues form different hairpins, P3 and P3′, in HP and PK, respectively (Fig. 1). Additionally, a polypyrimidine tract occurs at nucleotides 696 to 702, which form the 3′ half of hairpin P1. In the absence of multivalent cations P1 is extremely reactive, including stem positions, indicating that P1 may be particularly unstable (Fig. 3B). Roles for splice site RNA structure in regulating splicing, e.g. by hiding and revealing splice sites or protein binding sites, have been described for other RNAs [48]. In particular, RNA conformational switching can be induced by proteins [49], [50] or small molecules [51], [52] to regulate splicing. Expression of M2 protein is known to occur late in viral infection [15]. Perhaps changes in the cellular environment over time: such as pH, protein binding, or the presence of metabolites could affect the 3′ splice site structure to make this region more accessible and contribute to increased production of M2 mRNA.

Increased Mg^2+^ allows for some pseudoknot folding, but even 25 mM Mg^2+^ cannot push the equilibrium completely to pseudoknot (Fig. 2A). Addition of [Co(NH_3_)_6_]^3+^, however, preferentially stabilizes the 3PSS pseudoknot (Fig. 2B). Preferable binding of [Co(NH_3_)_6_]^3+^ to pseudoknots over hairpins by 3 to 5 fold has been described [53], [54]. Compared to hexahydrated magnesium, [Co(NH_3_)_6_]^3+^ has a similar size but a higher charge density, allowing for stronger interactions with RNA [55], [56]. The particular affinity of [Co(NH_3_)_6_]^3+^ to pseudoknots has been attributed to the tendency of pseudoknots to have pockets of high negative charge at the intersection of adjacent pseudoknot helices and/or connecting loops [53]. NMR studies on a viral frameshift pseudoknot showed that [Co(NH_3_)_6_]^3+^ binds tightly to a divalent metal binding pocket formed by a two nucleotide loop and the major groove of a nearby helix [54].

Cobalt hexamine is also able to bind motifs containing GA pairs and can do so more strongly than Mg^2+^ [57]. Interestingly, one of the distinguishing features of the 3PSS pseudoknot is the possibility of forming multiple GA pairs in P3′: a single GA within the P3′ helix, and three tandem GA pairs in the terminal loop (Figs. 3A and 5A). When mutations occur in this loop, they are most often to another purine base (Fig. 5A and Table S2). Evidence for the strength of the interaction between PK and [Co(NH_3_)_6_]^3+^ can be inferred from the strong stops observed in primer extension on 3PSS folded in [Co(NH_3_)_6_]^3+^ (Fig. S2 lanes 5 to 8). Even though samples were washed in 70% ethanol and heated to 90**°**C, the reverse transcriptase is unable to read through the strong secondary structure of P3′ stabilized by the remaining [Co(NH_3_)_6_]^3+^.

### Implications from Results of Mapping RNA Structures

P1 is more accessible to enzymes in HP and HPMut than in PK. Even at low RNase I_f_ concentrations P1 is reactive in HP (Fig. 3B and Fig. S3 lane 5). At medium enzyme concentration, HP is extremely reactive in the P1 region (Fig. S3 lane 4). Conversely, P1 of PK is not strongly reactive, even in the loop region, at the highest enzyme concentration (Fig. 3A and Fig. S3 lane 15). Additionally, the loop of P1 in PK is not strongly reactive with RNase A and T1, but is strongly reactive in HPMut (Fig. 3). This difference is unlikely to arise from changes in buffer: HPMut folded in 5 mM [Co(NH_3_)_6_]^3+^ also has a strong RNase A and T1 cleavage in the P1 loop (Fig. S1C). HP is extremely reactive to RNAse A in the P1 region: at even the lowest enzyme concentration it is mostly degraded (Fig. S3 lane 8). These results indicate that P1 might be less accessible to proteins, such as splicing factors, in the pseudoknot conformation, but more accessible in the hairpin structure. P1 is expected to be less stable in HP than in PK because the ends are unrestricted upon P1 unfolding in HP. This provides a more favorable entropy change for unfolding in HP than in PK. In general, an open, flexible, polypyrimidine tract is better able to bind splicing factors [49], [58]. Interestingly, P1 of HPMut is reactive to RNase V1, which cleaves double stranded or stacked RNA, and also to reagents that are specific to single stranded RNA (Fig. S1D). In particular, U701 is strongly hit by both RNAse I_f_ and V1. These results suggest that the base pairs in P1 of HPMut are dynamic and may be “breathing.” This suggests the same dynamics for HP, although this cannot be directly proven because V1 requires Mg^2+^.

A striking feature of the Pb^2+^ cleavage results (Fig. 4) is the dramatic difference in reactivity for PK compared to HP and HPMut. In general, PK is much less reactive to Pb^2+^, except in J1/2. This effect does not appear to be due to competition between Pb^2+^ and the [Co(NH_3_)_6_]^3+^ used to fold PK; HPMut, when folded in 5 mM [Co(NH_3_)_6_]^3+^ has wide-spread reactivity (Fig. S1C) that is more similar to HP in 100 mM KCl. The strongest Pb^2+^ cleavages are in the J1/2 regions of PK and HPMut: specifically at A704 and A705 of PK and HPMut, respectively (Fig. 4B). J1/2 of HP is also sensitive to Pb^2+^ cleavage, yet there are no cleavages as intense as in PK or HPMut. Perhaps when the RNA is folded in the absence of multivalent cations (HP) a specific binding pocket for lead, such as in tRNA [59] and in a group I intron [60], may no longer form. DEPC modifies the N7 position of adenosine, which can participate in tertiary contacts. Notably, J1/2 in HP is strongly reactive to DEPC but J1/2 is not strongly reactive in PK and HPMut where multivalent ions are present. This provides further evidence for a potential tertiary fold for J1/2.

Except for the RNase T1 hit at G723, the loop of P3 in HP and HPMut is insensitive to enzymes, but quite reactive with small molecules (Fig. 3B and C and Fig. S1B, C, and D). Reactivity in the P3 helix is confined to the bulge loop at A730, and a single DMS hit at A719 of HPMut, where A719 is paired, but in a weak structural context. In contrast, P3′ is very sensitive to enzyme cleavage (Fig. 3A and Fig. S1A). An unusual feature of P3′ is that the greatest reactivity occurs at the 5′ side of the hairpin and includes three residues involved in base pairs (Fig. 3A). P3′ has fewer stretches of canonical pairs than any other region of PK as well as several putative non-canonical pairs (Fig. 5A). P3′ may be structurally dynamic. Indeed, breaking base pairs in P3′ would be necessary to transition to the hairpin conformation. This structure may be tuned to easily open and facilitate conformational switching.

It may be possible to target either or both of the conformations of 3PSS with short oligonucleotides in order to modulate biological function or for potential therapeutic applications. The results of oligonucleotide microarray mapping show that the hybridization behavior of PK, HPMut and HP are distinct (Fig. 3, Fig. S1, and Table S1). Though the binding results are influenced by buffer conditions, which are not physiological, the strong binding centers for the probes suggest regions for targeting with oligonucleotides. The small hexamer and pentamer binding sites may be used as nucleation sites for the binding of larger oligonucleotides such as siRNAs [61] or shRNAs [62].

### Conservation of Structure

The HP and PK conformations are well conserved throughout influenza A strains. They span a region that is under strict selective pressure: it must maintain open reading frames for M1 and, after nt 714, the M2 protein open reading frame, as well as protein binding sites [11]. Mutations must also maintain RNA secondary structure (Fig. 5). Conservation is also favored by the influenza encoded polymerase, which has higher fidelity than most viral polymerases [63]. The high conservation of structure in the region containing the 3′ splice site of segment 7 makes this region an attractive therapeutic target. Moreover, the presence of small loops favors approaches for rational selection of molecules as lead compounds [28], [29], [31]. The microarray results (Fig. 3 and Table S1) suggest that short oligonucleotides may also be used to inhibit correct splicing. Similarly, the M1 protein amino acids encoded by 3PSS may be attractive targets for antiviral agents, as their evolution is also strictly constrained by the need to maintain functional sites and structure in both the protein and RNA.

Mutations from canonical to non-canonical pairs in the 3PSS region are rare (Table S2). When they occur they are in the middle of stems, where they might be less disruptive to structure. Indeed, only two types of non-canonical pairs have significant numbers at sites predominantly canonically paired (Table S2): CA pairs, which can maintain A form helices [64], followed by GA pairs, which can substitute for canonical pairs in phylogenetically conserved structures [65], [66], [67]. Conversely, at sites where putative non-canonical pairs predominate, mutations occur most often to form canonical pairs or other putative non-canonical interactions (Fig. 5 and Table S2). For example: in P3′, nucleotides 719 and 734 are most often AG, but the most frequently observed mutation converts this to a UG pair (Fig. 5A and Table S2). Imino AG and canonical UG pairs both present an amino group in the minor groove, which can be used for molecular recognition [68], [69], [70]. Three consecutive GA pairs are possible in the terminal loop of P3′ (Fig. 5A). When mutations occur they maintain purines at each side of the helix. This sequence pattern is common in internal loops, where it results in three consecutive purine-purine sheared pairs [71]. In HP the two-by-two nucleotide internal loop may contain non-canonical interactions as well. G736 frequently changes to a U, allowing it to pair with G714 (Fig. 5B) and positions 715 and 735 are always purines: GA or GG, which have been observed to form interactions in other RNAs [72], [73]. Interestingly, internal loops comprised of GG and GA are observed in the ribosomal loop E motif [74] and in the HIV-1 Rev protein binding element [75]; in both cases the loop plays important roles in protein recognition.

Another conserved feature in the structural model and alignment (Fig. 5A and Table S4) is the possibility of forming base triples at G687(C712-G738), U686(A713-U737) and (C685-G714)G736 in PK. UAU and CGG are the most common base triples in known 3D structures of RNA [76]. Sequence variations at these positions could maintain potential triple interactions (Table S4). Because of the close proximity of stems and loops, such loop-helix interactions are commonly found in pseudoknots [77], [78], [79] and can play important roles in stabilizing structure [80]. These putative triples occur at the intersection of the P0 and P2 pseudoknot helices, which may form a coaxial stacking interaction where G714 is stacked on A713 and U737 is stacked on C685 (Fig. 5A). The same type of stacking interaction, where pseudoknot helices coaxially stack with AU on CG, is observed in a bacteriophage mRNA pseudoknot [81], [82].

#### Host species distribution of number of canonical pairs and GC pair content

3PSS sequences segregated into five groups based on their canonical base pair and GC pair content. The composition of each cluster varied dramatically by the host specificity of the influenza A strain (Fig. 6): the greater the strength of the structure, as gauged by the overall base pair density and number of GC pairs, the higher the fraction of avian vs. human strains. The swine specific strains fell somewhere between human and avian. These trends may be explained by the temperatures encountered where influenza replicates. Temperatures for the avian gut, swine and human respiratory tract are 41°C, 37°C, and 33°C, respectively [83]. Perhaps the higher number of canonical and GC pairs in avian strains occurs to maintain the pseudoknot and hairpin structures, or their ratio, at higher temperatures. Conversely, at the lower temperatures found in swine and human hosts, there is less pressure to stabilize these structures. The host specificity of the observed changes in the 3′ splice site region may be a local instance of a global trend in influenza A RNA structural stability. A study of all available influenza A coding regions found that there were global trends in RNA folding free energy and, in general, avian sequences were more stable than swine or human [84]. Additionally, four of the eight influenza A segments, including segment 7, showed evidence for globally conserved RNA secondary structure [84]. Interestingly, the strength of this global structure also favored avian sequences.

## Methods

### Production of 3PSS and HPMut RNAs

DNA templates for 3PSS and HPMut RNAs, including T7 promoter sites were ordered from IDT Inc. The 3PSS sequence was selected from the GC pair rich cluster and is found in four avian sequences of mixed strains (GenBank accessions: CY081301, CY021470, CY014592, DQ107463). HPMut has an identical sequence except where mutations were introduced to abolish the pseudoknot: positions 684-6 (mutated to unpaired adenosines) and 716-7/733-4 (two hairpin CG pairs swapped to GC). *In vitro* transcription reactions were performed using an *Ampliscribe T7-Flash Transcription* kit (Epicentre). Products were purified by denaturing PAGE and electroeluted in a Bio-Rad Model 422 electro-eluter. RNAs were 5′ end labeled with γ-^32^P ATP (Perkin Elmer), then re-purified by denaturing PAGE.

### RNA Folding for Native Gel Analysis

For each sample, about 100,000 cpm of 5′ end labeled RNA was heated in water to 90°C for 2 min and slowly cooled to 50°C in a thermocycler. Tris and KCl were added to a final concentration of 10 mM Tris (pH 7) and 100 mM KCl, at 50°C, for all samples. To study the multivalent cation dependent folding of 3PSS, MgCl_2_ was added to get a range of final concentrations from 2.5 to 25 mM and, in separate samples without Mg^2+^, [Co(NH_3_)_6_]Cl_3_ was added to span 0.002 to 5 mM. HPMut was folded with a final concentration of 10 mM MgCl_2_ or 5 mM [Co(NH_3_)_6_]^3+^ at 50°C. All samples were then slow cooled from 50°C to 37°C where they remained for 15 min before placing them on ice. Folding was analyzed by native gel electrophoresis. Glycerol loading buffer (3 µL) was added to each sample and about 20,000 cpm of folded RNA (2 µL) was run per lane on a non-denaturing 6% polyacrylamide gel made with 1X THEM (34 mM Tris Base, 57 mM HEPES, 0.1 mM EDTA, 10.0 mM MgCl_2_) buffer. The gel was run using 1×THEM running buffer, at low Wattage (15 W), at 4°C in order to maintain folding [85]. After 6.5 h, the gel was dried, exposed to a phosphorscreen, and imaged using a Bio-Rad Personal Molecular Imager.

### Chemical and Enzymatic Mapping

RNAs used in all mapping experiments were folded as described for native gel analysis. Each sample had a final buffer and monovalent ion composition of 10 mM Tris (pH 7) and 100 mM KCl. HPMut contained 10 mM Mg^2+^ or 5 mM [Co(NH_3_)_6_]^3+^, PK 5 mM [Co(NH_3_)_6_]^3+^ and HP no multivalent ions. Enzymatic and small molecule mapping was carried out at room temperature.

RNase I_f_, A, T1, and V1 reactions, alkaline hydrolysis and RNase T1 ladder were adapted from manufacturer’s protocol (Ambion, Inc and New England Biolabs) and carried out on 5′ end labeled RNAs (50,000 cpm per reaction). Optimal enzymatic concentrations were determined with enzyme titrations. The digestion reactions were stopped by ethanol precipitation at −20°C. The resulting pellet was dissolved in gel loading buffer and fractionated on a denaturing, 8% polyacrylamide gel.

Pb^2+^ cleavage reactions were carried out by incubating 5′ end labeled RNAs with 1 mM Pb(OAc)_2_ [86], [87]. Aliquots (50,000 cpm per aliquot) were removed at 0, 0.5, 1, 2, 4, 8, 15, 30, and 60 min. The reaction was stopped by placing the aliquots in gel loading buffer and freezing at −80°C until they were fractionated on a denaturing, 8% polyacrylamide gel. DEPC reactions were carried out by incubating 5′ end labeled RNAs (50,000 cpm per reaction) with 0.69 mM DEPC, followed by NaBH4 reduction and aniline cleavage. Reactions were stopped by precipitation at −20°C and the resulting pellet was dissolved in gel loading buffer and fractionated on a denaturing, 8% polyacrylamide gel.

Unlabeled 3PSS RNA (0.5 µg per reaction) was modified with optimized concentrations of DMS, CMCT, and NMIA using published protocols [87], [88]. Modifications were read out by primer extension (primer sequence: 5′-ACATCTGCACTCCC-3′, chemically synthesized by IDT, and 5′ end labeled with γ-^32^P ATP) with 100, 000 cpm per reaction, followed by separation of fragments by denaturing 8% PAGE.

All gels were dried, exposed to phosphorscreen, and imaged with a Bio-Rad Personal Molecular Imager. Gel images were analyzed with ImageJ [89]. Bands were quantified by taking the integrated pixel density and normalizing with respect to the highest intensity band after subtraction of background observed in negative controls, which were treated as for reactions, but with omission of the modifying reagent. For Pb^2+^ cleavage, equal amounts of radioactivity were loaded in each lane and so all bands were normalized to A704 of PK, which was the most intense in any gel.

### Hybridization to Oligonucleotide Microarrays

About 200,000 cpm of labeled RNA was folded as described above before hybridization to oligonucleotide microarrays [32], [38], [90], [91]. Each of the 861 probes was spotted in triplicate. Spotting buffer, monomer U, and pentamer UUUUU, which should show no binding to 3PSS, were also printed on the microarray as internal negative controls. Hybridizations were carried out in folding buffer for 18 h at 4°C. Microarrays were washed for 1 min at 0°C and then dried by centrifugation. Hybridization was visualized by exposure to a phosphorimager screen and quantitative analysis was performed with ArrayGaugeV2.1. Binding was considered strong, medium and weak, when the integrated intensities were ≥1/3, ≥ 1/9 and ≥ 1/27 of the strongest integrated intensity, respectively. Alternative binding sites were predicted using RNA-RNA thermodynamics [92], [93].

### Sequence Alignment and Analysis

All full-length, non-redundant influenza A sequences (13,277) were downloaded from the National Center for Biotechnology Information (NCBI) influenza virus resource page [94]. An alignment was generated with the MAFFT alignment algorithm’s FFT-NS-1 method [95]. The 3PSS region was cut from the large alignment and sequence duplicates were collapsed using a PERL script to identify 734 unique sequences. The nucleotide alignment was converted to amino acids *in silico*, re-aligned with ClustalW [96], manually refined, then converted back into nucleotides. Base pairing frequencies from each model (Fig. 3A,B) were analyzed with respect to the whole alignment and each unique sequence using PERL scripts.

Free energies at 37°C were predicted for the hairpin and pseudoknot conformations with a nearest neighbor thermodynamic model [92], [93], [97], [98] and pseudoknot loop entropy model [99].

## Supporting Information

Figure S1 **Results of experimental mapping.** Specific reagent is indicated by colored shapes (see figure key). Reactivity ≥2/3 the strongest band is indicated with solid shapes, while reactivity <2/3 but ≥1/3 the strongest band is indicated by open shapes. All folding buffers contained 10 mM Tris (pH7), 100 mM KCl. Mapping results for: (**A**) 3PSS folded in 5 mM [Co(NH_3_)_6_]^3+^ (PK). (**B**) 3PSS folded without Mg^2+^ or [Co(NH_3_)_6_]^3+^ (HP) RNase A reactivity is not annotated because RNA is over-digested at the same enzyme concentration that yielded good results in PK and HPMut. (**C**) HPMut folded in 5 mM [Co(NH_3_)_6_]^3+^, and mapped with Pb^2+^ and RNases A and T1. (**D**) HPMut folded in 10 mM Mg^2+^. (**E**) 3PSS folded in 10 mM Mg^2+^ which gives roughly equal amounts of PK and HP. Results are annotated on both structure models (SHAPE mapping was not performed on the mixture). RNase V1 was only used when folding conditions contained Mg^2+^, which is essential to enzyme activity [82]. Dark and light orange letters represent strong and moderate RNase V1 hits.
(TIF)

Figure S2 **Gel results for primer extension.** Readouts of DMS, kethoxal, and CMCT experiments on pseudoknot (PK), hairpin (HP), and hairpin mutant (HPMut) are shown. The first four lanes are dideoxy ladders and the remaining are for experiments on each RNA target. Unmodified RNA experimental controls (Exp. Control) were run alongside each set of experiments to show natural stops induced by target structure. Interpretable primer extension data for PK stretches from nts 683 to 715.
(TIF)

Figure S3 **Gel results for enzymatic mapping experiments on HP and PK.** T1 ladders and hydrolysis ladders (OH ladders) are run alongside mapping lanes to identify cleavage sites. For each enzyme the black wedge indicates the increasing range of enzyme used: RNase I_f_ had 50 U, 5 U, 0.5 U; RNase A had 1 ng, 0.1 ng and 0.01 ng; and RNAse T1 had 1 U, 0.1 U, and 0.01 U. The last lane is an experimental control for RNA treated the same as enzyme reactions, but without any enzyme.
(TIF)

Table S1 **Table of results for microarray hybridization experiments.**
(XLSX)

Table S2 **Base pairing frequencies and percent canonical pairing**. Pseudoknot (PK) and hairpin (HP) conformation counts based on an alignment of unique sequences and also all available sequences. Data for PK and the helixes P1 and P2 in HP are shown in the top box. The P3 helix of HP is shown in the bottom box. Paired sites are indicated by the i and j locations of the 5′ and 3′ nts. Symmetric loop sites are given in italics. Helixes are colored purple for P0, orange for P1, green for P2, and blue for P3/P3′. Mutations expected to be compensatory (double point mutations that preserve pairing) are annotated in blue. Mutations expected to be consistent (single point mutations that maintain pairing) are annotated in green. The last column gives the percentage of the time i and j are canonically paired in the alignment of all unique sequences. Also included are the base pair type percentages by helix and averaged across helices.
(XLSX)

Table S3 **Table with GenBank accession numbers and sequences used in this study.** Unique sequences are ranked according to their ability to form stable secondary structure. The most stable are at the top, while the least stable are at the bottom. Relative stability is estimated as the fraction of GC pairs multiplied by the fraction of nucleotides in canonical pairs (color annotated red to green for both PK and HP). Also shown are the number of sequences that collapse into each unique sequence, the host specificity and the viral strain.
(XLSX)

Table S4 **Counts for putative base triples.** The most frequent triple is in red and the second most frequent in orange.
(XLSX)
