# Correction: The 3′ Splice Site of Influenza A Segment 7 mRNA Can Exist in Two Conformations: A Pseudoknot and a Hairpin

**DOI:** 10.1371/annotation/b3e4a549-7b87-41ac-a9a7-78d2e77ea7a5

**Published:** 2012-09-18

**Authors:** Walter N. Moss, Lumbini I. Dela-Moss, Elzbieta Kierzek, Ryszard Kierzek, Salvatore F. Priore, Douglas H. Turner

The following information was missing from the Funding section: This work was also supported by the National Institute of Health grant 1R03TW008739-01 (E.K. and D.H.T), and National Science Center grant N N301 788 440 (E.K.). 

