# Supplementary figures and images for: The 3′ Splice Site of Influenza A Segment 7 mRNA Can Exist in Two Conformations: A Pseudoknot and a Hairpin
Source: PLoS One. 2012 Jun 7;7(6):e38323. doi: 10.1371/journal.pone.0038323 (PMC3369869; doi:10.1371/journal.pone.0038323)

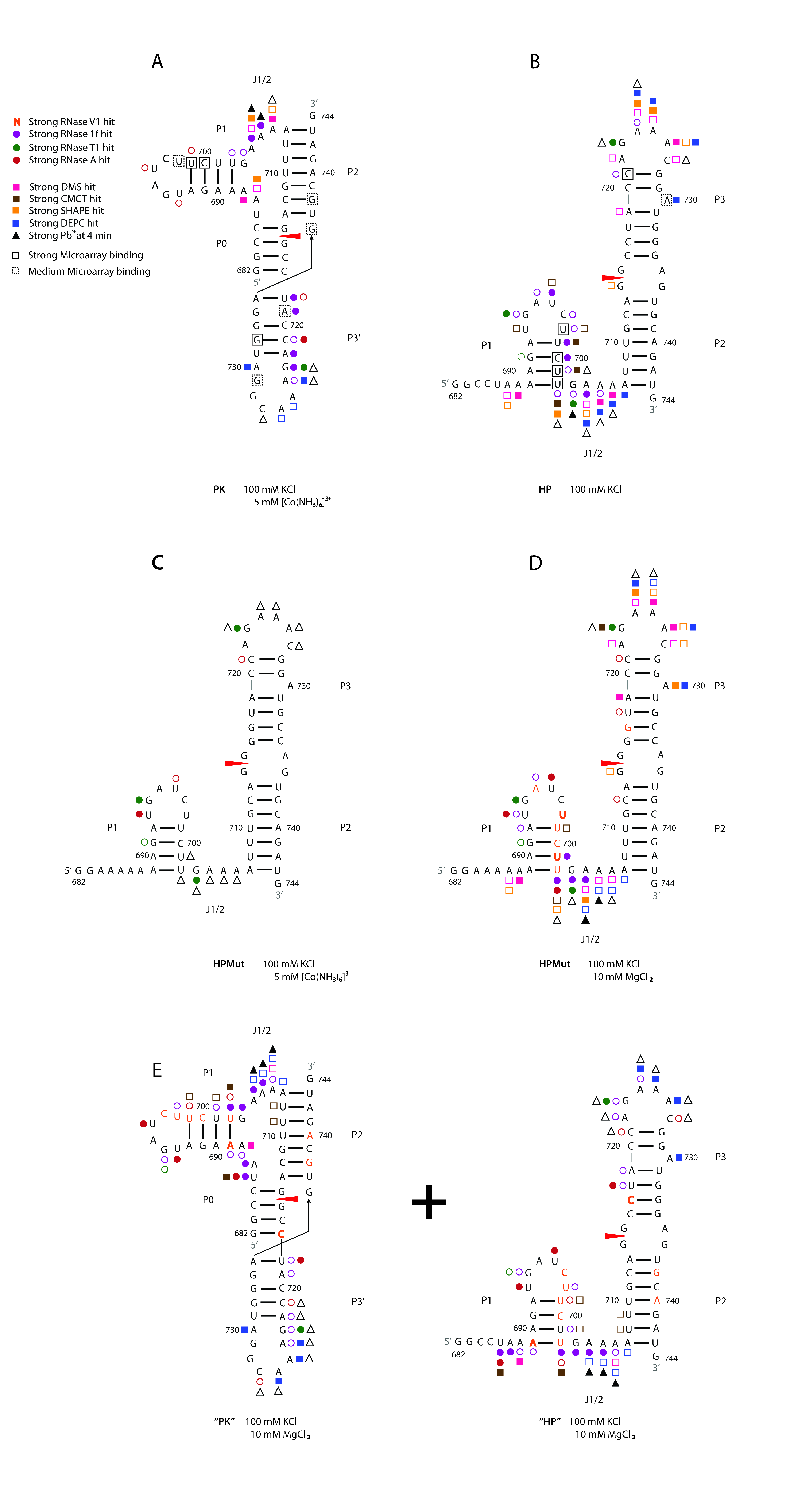

Supplement: Figure S1 — Results of experimental mapping. Specific reagent is indicated by colored shapes (see figure key). Reactivity ≥2/3 the strongest band is indicated with solid shapes, while reactivity <2/3 but ≥1/3 the strongest band is indicated by open shapes. All folding buffers contained 10 mM Tris (pH7), 100 mM KCl. Mapping results for: (A) 3PSS folded in 5 mM [Co(NH3)6]3+ (PK). (B) 3PSS folded without Mg2+ or [Co(NH3)6]3+ (HP) RNase A reactivity is not annotated because RNA is over-digested at the same enzyme concentration that yielded good results in PK and HPMut. (C) HPMut folded in 5 mM [Co(NH3)6]3+, and mapped with Pb2+ and RNases A and T1. (D) HPMut folded in 10 mM Mg2+. (E) 3PSS folded in 10 mM Mg2+ which gives roughly equal amounts of PK and HP. Results are annotated on both structure models (SHAPE mapping was not performed on the mixture). RNase V1 was only used when folding conditions contained Mg2+, which is essential to enzyme activity [82]. Dark and light orange letters represent strong and moderate RNase V1 hits. (TIF) [file pone.0038323.s001.tif]

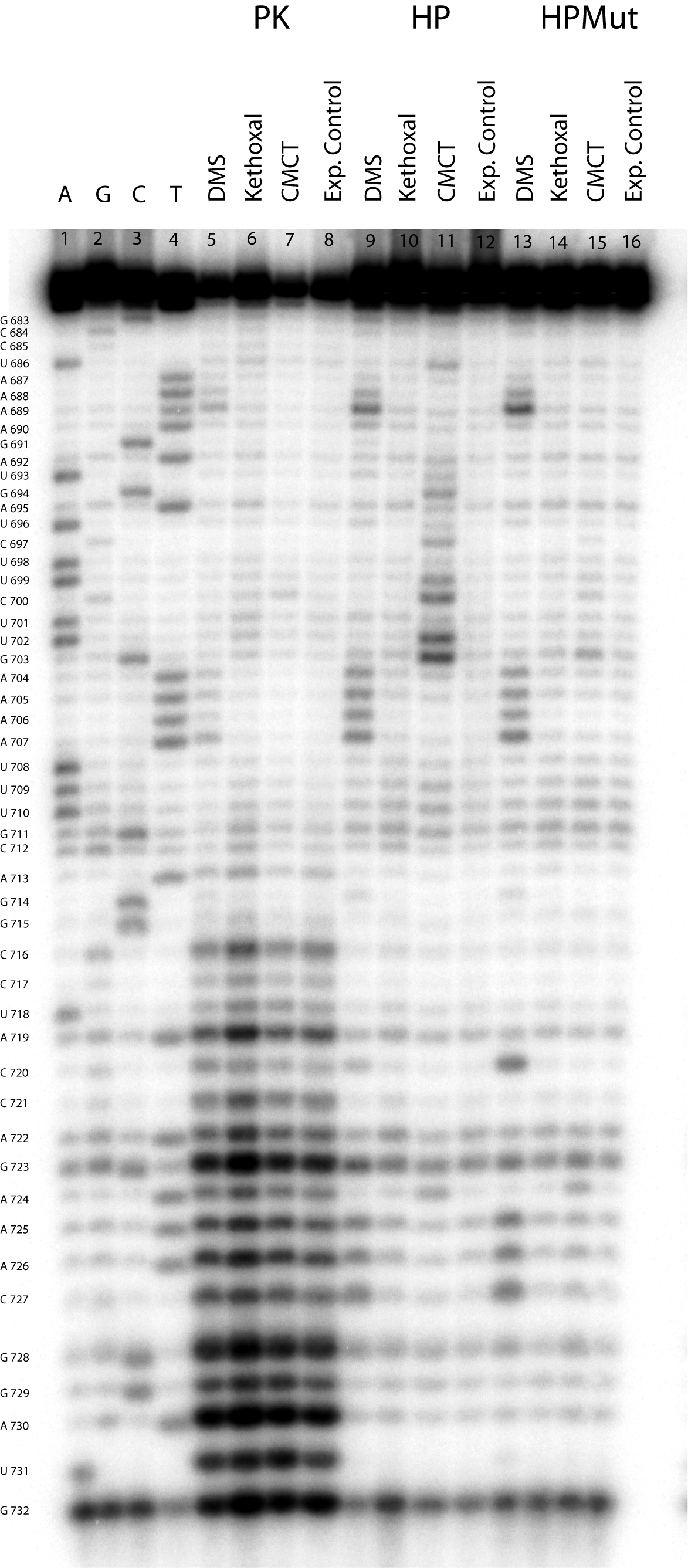

Supplement: Figure S2 — Gel results for primer extension. Readouts of DMS, kethoxal, and CMCT experiments on pseudoknot (PK), hairpin (HP), and hairpin mutant (HPMut) are shown. The first four lanes are dideoxy ladders and the remaining are for experiments on each RNA target. Unmodified RNA experimental controls (Exp. Control) were run alongside each set of experiments to show natural stops induced by target structure. Interpretable primer extension data for PK stretches from nts 683 to 715. (TIF) [file pone.0038323.s002.tif]

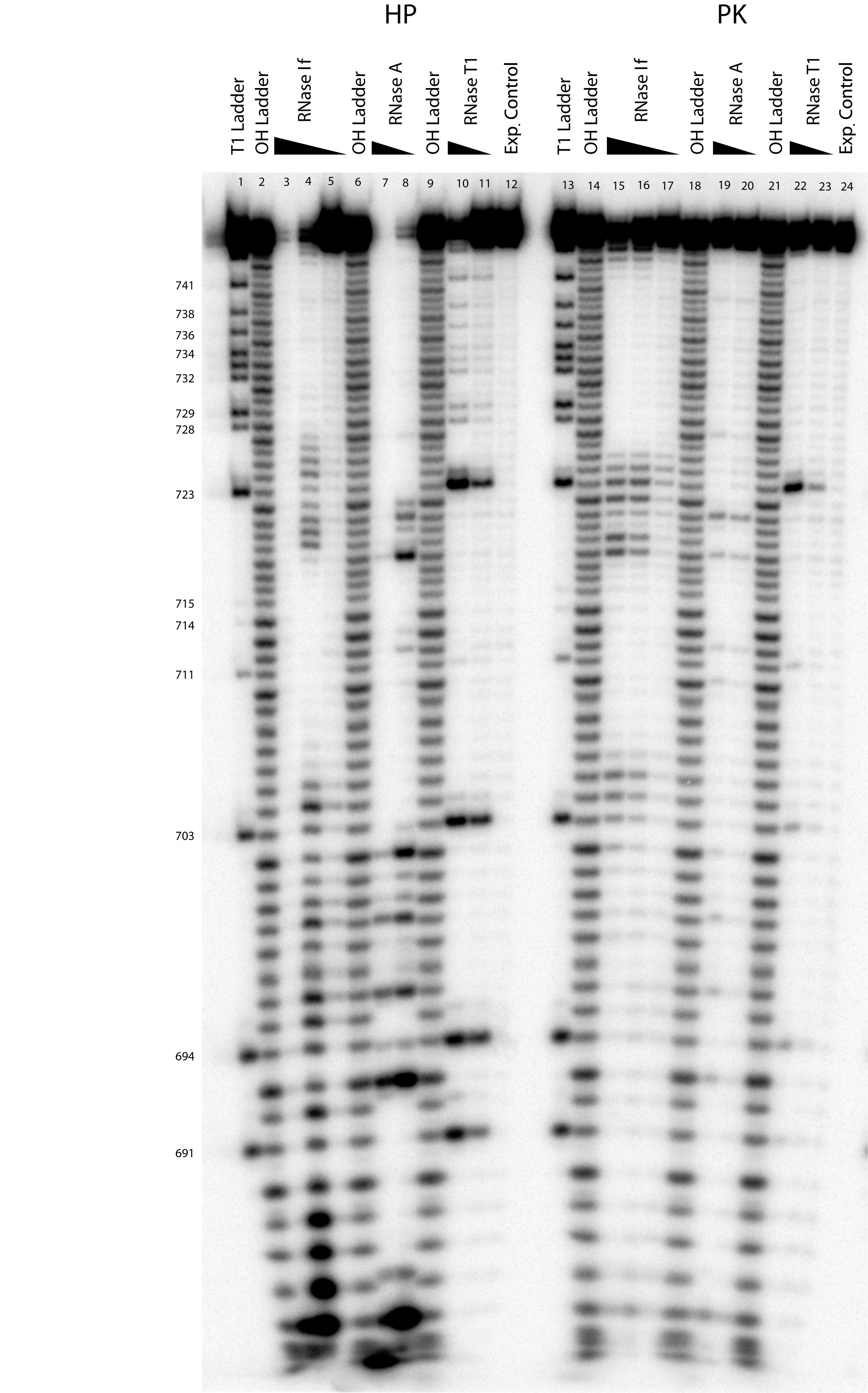

Supplement: Figure S3 — Gel results for enzymatic mapping experiments on HP and PK. T1 ladders and hydrolysis ladders (OH ladders) are run alongside mapping lanes to identify cleavage sites. For each enzyme the black wedge indicates the increasing range of enzyme used: RNase If had 50 U, 5 U, 0.5 U; RNase A had 1 ng, 0.1 ng and 0.01 ng; and RNAse T1 had 1 U, 0.1 U, and 0.01 U. The last lane is an experimental control for RNA treated the same as enzyme reactions, but without any enzyme. (TIF) [file pone.0038323.s003.tif]
